# The Impact of Transportation Mode on ST-Segment Elevation Myocardial Infarction (STEMI) Reperfusion Time and Survival

**DOI:** 10.7759/cureus.101483

**Published:** 2026-01-13

**Authors:** Zeyad K Aljaili, Ahmed A Alsalman, Abdulrahman G Aljabri, Rayyan O Turkestani, Mohammed H Alghanim, Fatima H Al Saeed, Bader K Alossaimi, Dunya Alfaraj

**Affiliations:** 1 College of Medicine, Imam Abdulrahman Bin Faisal University, Dammam, SAU; 2 Emergency Department, Imam Abdulrahman Bin Faisal University, King Fahad University Hospital, Dammam, SAU

**Keywords:** door-to-balloon (d2b) time, door-to-ecg time, emergency medical services (ems), primary percutaneous coronary intervention (pci), st-segment elevation myocardial infarction

## Abstract

Background: Acute coronary syndrome continues to be the leading cause of mortality worldwide, making it crucial to achieve a door-to-balloon (D2B) time under 90 minutes to enhance patient outcomes. This study aims to evaluate how outcomes differ between patients who arrive via emergency medical services (EMS) and those who arrive via self-transport.

Methods: This was a retrospective study conducted at a tertiary hospital in the eastern province of Saudi Arabia. The study included both Saudi and non-Saudi patients who presented to the emergency department (ED) between March 1, 2022, and November 11, 2024, with ST-segment elevation myocardial infarction (STEMI) and non-ST-segment elevation myocardial infarction (NSTEMI) requiring primary percutaneous coronary intervention (PCI). Patients were divided into two groups based on their mode of transport. Collected data include age, gender, nationality, D2B and door-to-electrocardiogram (ECG) times, smoking status, chronic conditions, and peak troponin levels.

Results: A total of 204 STEMI and NSTEMI infarction cases were included in this study. Most cases (172, 84.3%) arrived via self-transport, while 32 (15.7%) arrived via EMS. The EMS group demonstrated significantly shorter median times for both door-to-ECG time (3 minutes vs. 5.5 minutes; p<0.05) and D2B time (70 minutes vs. 87 minutes; p<0.05) compared to the self-transport group. In addition, the EMS group had a higher incidence of cardiopulmonary resuscitation (CPR), indicating that their initial presentation is likely to be critical.

Conclusion: Our findings are consistent with existing literature, highlighting the role of EMS in reducing door-to-ECG and D2B times. The higher frequency of CPR in EMS-transported patients indicates the severity of their condition upon arrival, further emphasizing the importance of prehospital emergency care in optimizing outcomes.

## Introduction

Globally, acute coronary syndrome remains the leading cause of mortality, with around 50% of these fatalities taking place prior to admission [1,2]. Early reperfusion is essential for individuals with ST-segment elevation myocardial infarction (STEMI) when readily available [3]. In addition, having a door-to-balloon (D2B) time of less than 90 minutes is established as a quality metric for evaluating the efficacy of hospital systems in managing STEMI treatment, and delays in D2B are associated with increased morbidity and mortality [3-6]. Despite this increased emphasis, just about a third of patients in the United States have a percutaneous coronary intervention (PCI) within 90 minutes [7,8].

A factor influencing D2B time is the mode of transport to an emergency department (ED), whether via an emergency medical services (EMS) or personal transportation. Studies demonstrate that patients with STEMI who self-transport experience prolonged D2B periods compared to those transported by EMS [9,10]. Studies have also demonstrated a shorter interval between hospital arrival and symptom onset [11,12]. Prehospital care administered by EMS plays a critical role in improving access to treatment and reducing mortality in patients with STEMI [13-17]. Due to the utilization of EMS transport, accelerated and efficient treatment decisions are made, as EMS personnel are capable of conducting an electrocardiogram (ECG) and notifying the hospital of the patient's arrival, consequently reducing door-to-reperfusion times [18-22]. Nonetheless, evidence indicates that EMS is not being utilized to its full potential and a considerable number of STEMI patients continue to present at the ED using personal transportation [12,23].

To the best of our knowledge, no studies in Saudi Arabia have explored how the mode of transport affects D2B and other outcomes in patients with myocardial infarction (MI). This involves an examination of the transportation method employed by patients to reach the emergency room, alongside an analysis of the subsequent patient outcomes, which encompass the duration of hospital stay and mortality rate. This gap in the literature highlights an important area for future investigation and underscores the need for targeted studies to enhance our understanding of this aspect of patient care.

## Materials and methods

Ethical approval and consent to participate

The Imam Abdulrahman Bin Faisal University Institutional Review Board (IRB) granted permission to this study (approval number: IRB-2024-01-719). In addition, we obtained approval from the catheterization lab to view door time, ECG time, and balloon inflation time for each participant. Since no personally identifiable information was obtained, informed consent was not necessary. The Declaration of Helsinki was adhered to throughout the study.

Study design

This retrospective study was performed at King Fahad University Hospital, a tertiary hospital in the eastern province of Saudi Arabia. We gathered the following data from the hospital's record system in the catheterization lab: (1) age at presentation, (2) gender, (3) time, (4) door-to-ECG time, (5) length of stay in days, (6) smoking status, (7) chronic conditions (diabetes mellitus, hypertension, and dyslipidemia), (8) peak troponin levels, which correlate with the severity of the infarction, (9) whether the patient suffered an episode of ventricular fibrillation/ventricular tachycardia, (10) whether cardiopulmonary resuscitation (CPR) was administered, (11) ejection fraction post-PCI, and (12) if the patient had PCI done on a prior presentation.

Study subjects and inclusion and exclusion criteria

Our study includes Saudi and non-Saudi patients presenting to the ED between March 1, 2022, and November 11, 2024, with MI requiring primary PCI. Patients meeting the criteria of ST-segment elevation (ST-segment elevation at least 2 adjacent precordial leads on admission ECG and ≥0.20 mV in leads V2 and V3 and ≥0.10 mV in other leads) or STEMI equivalent were included in our study. We excluded non-ST-segment elevation myocardial infarction (NSTEMI) and STEMI equivalents if PCI was not done. Because door-to-ECG and D2B times are prospectively captured as catheterization laboratory performance metrics only for primary PCI cases, this retrospective study was inherently limited to patients undergoing primary PCI.

Variables

The independent variables in this study were age, gender, nationality (given its potential influence on access to healthcare through communication barriers and socioeconomic factors), mode of transport, smoking status, and the presence of chronic conditions. The dependent variables were the D2B and door-to-ECG times, peak troponin levels, length of hospital stay, whether CPR was administered or not, if the patient suffered an episode of ventricular fibrillation/ventricular tachycardia, and ejection fraction post-PCI.

Procedures and materials

Door time, ECG time, and balloon inflation time were originally documented manually on paper forms stored in the catheterization laboratory as performance metrics for each STEMI and NSTEMI patient requiring primary PCI. After obtaining the necessary approvals for research use, including IRB approval, catheterization laboratory approval, and IT authorization to access data from the hospital record system, these times were transcribed into a structured Excel sheet (Microsoft Corporation, Redmond, Washington, United States). Based on these times, D2B and door-to-ECG times were calculated. Additional clinical information, such as length of hospital stay, peak troponin levels, and mode of transportation, was extracted from patients' electronic medical records. 

Statistical analysis

In our study, participants were categorized into two groups: those who arrived by EMS and those who self-transported. Statistical analyses were conducted using IBM SPSS Statistics for Windows, Version 26.0 (IBM Corp., Armonk, New York, United States). The chi-squared test was applied for categorical baseline characteristic variables. D2B and door-to-ECG times were assessed for normality and reported as medians with interquartile ranges (IQR), with comparisons made using the Mann-Whitney U test. For in-hospital clinical outcomes, categorical variables were analyzed with the chi-squared test, while continuous variables were evaluated using the Mann-Whitney U test. Additionally, a multivariate logistic regression analysis was conducted to examine variables related to the mode of transport. A p-value of less than 0.05 was considered statistically significant for all analyses, and categorical variables were presented with frequencies and percentages.

## Results

**Figure 1:**
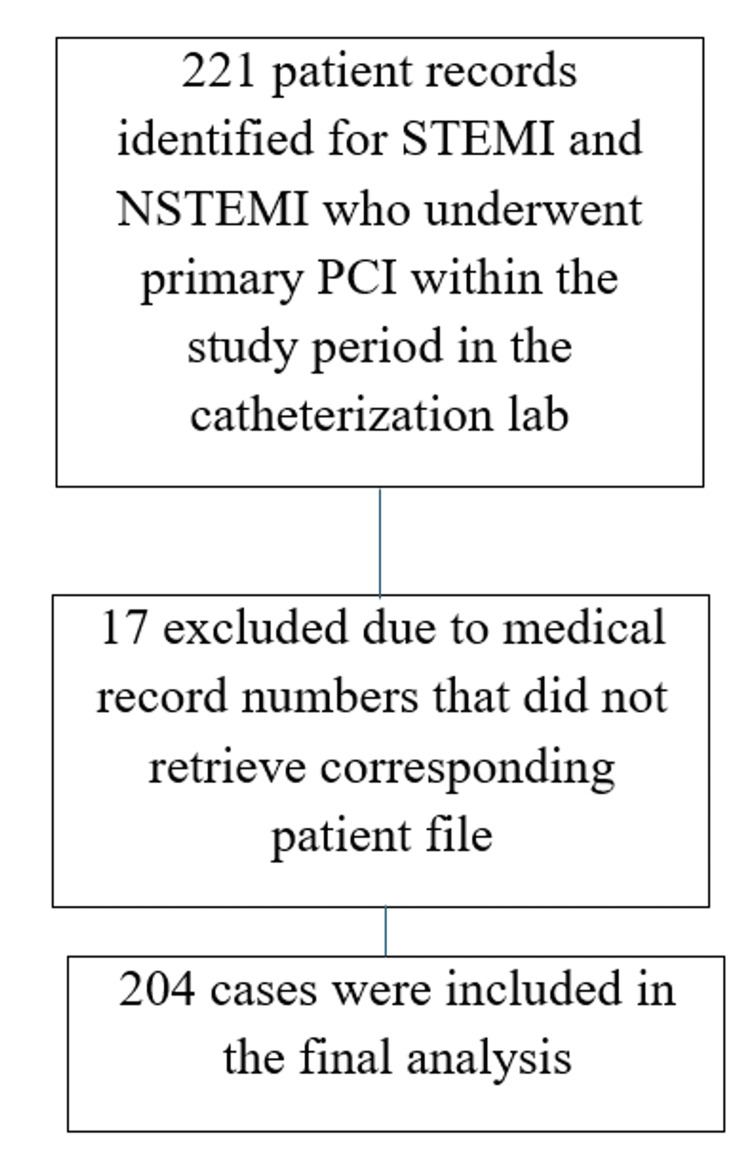
Patient selection and exclusion flow diagram STEMI: ST-segment elevation myocardial infarction; NSTEMI: non-ST-segment elevation myocardial infarction; PCI: percutaneous coronary intervention

A total of 204 STEMI and NSTEMI cases were included in this study. The majority of cases (172, 84.3%) arrived at the ED via self-transport, while 32 (15.7%) were transported by EMS. Figure 1 depicts the patient selection and exclusion flow diagram.

Table 1 compares the demographic characteristics of patients based on their mode of transport to the hospital: self-transport versus EMS. Males constituted 148 (86%) of self-transport patients and 31 (96.9%) of EMS patients, and females were 24 (14%) in the self-transport group and one (3.1%) in the EMS group. The difference was not statistically significant (p=0.08). Patients under 50 years old made up 66 (38.4%) of self-transport and nine (28.1%) of EMS patients. Patients aged 50 and above were 106 (61.6%) in the self-transport group and 23 (71.9%) in the EMS group. The difference was not statistically significant (p=0.27). Saudi nationals were 68 (39.5%) in the self-transport group and 12 (37.5%) in the EMS group. Non-Saudi nationals were 104 (60.5%) in the self-transport group and 20 (62.5%) in the EMS group. The difference was not statistically significant (p=0.82).

**Table 1:** Comparison of patient demographics between the EMS group and the self-transport group (n=204) EMS: emergency medical services

			Self-transport (n=172)	EMS (n=32)	P-value
Gender	Male		148 (86%)	31 (96.9%)	0.08
	Female		24 (14%)	1 (3.1%)	
Age (years)	<50		66 (38.4%)	9 (28.1%)	0.27
	≥50		106 (61.6%)	23 (71.9%)	
Nationality	Saudi		68 (39.5%)	12 (37.5%)	0.82
	Non-Saudi		104 (60.5%)	20 (62.5%)	

Table 2 compares the baseline clinical characteristics of patients based on their mode of transportation. The majority (21, 65.6%) of EMS patients were diabetic compared to self-transport patients (84, 48.8%), but the difference was not statistically significant (p=0.07). The proportion of hypertension was high in EMS patients (18, 56.3%), compared to self-transport patients (76, 44.2%), but the difference was not statistically significant (p=0.2). Dyslipidemia was also high in EMS patients (11, 34.4%), as compared to self-transport patients' cases (49, 28.5%). The difference was not statistically significant (p=0.49). The distribution of smokers was similar in both self-transport and EMS patients; 71 (41.3%) self-transport patients and 15 (46.9%) EMS patients were smokers (p=0.56).

**Table 2:** Comparison of patient baseline characteristics between the EMS group and the self-transport group (n=204) EMS: emergency medical services; PCI: percutaneous coronary intervention

		Self-transport (n=172)	EMS (n=32)	P-value
Diabetic		84 (48.8%)	21 (65.6%)	0.07
Hypertensive		76 (44.2%)	18 (56.3%)	0.2
Dyslipidemia		49 (28.5%)	11 (34.4%)	0.49
Smoker		71 (41.3%)	15 (46.9%)	0.56
PCI done before		38 (22.1%)	7 (21.9%)	0.91
Onset of pain	<2 hours	57 (33.1%)	12 (37.5%)	0.62
	2-6 hours	51 (29.7%)	9 (28.1%)	0.89
	>6 hours	33 (19.2%)	3 (9.4%)	0.18

**Figure 2:**
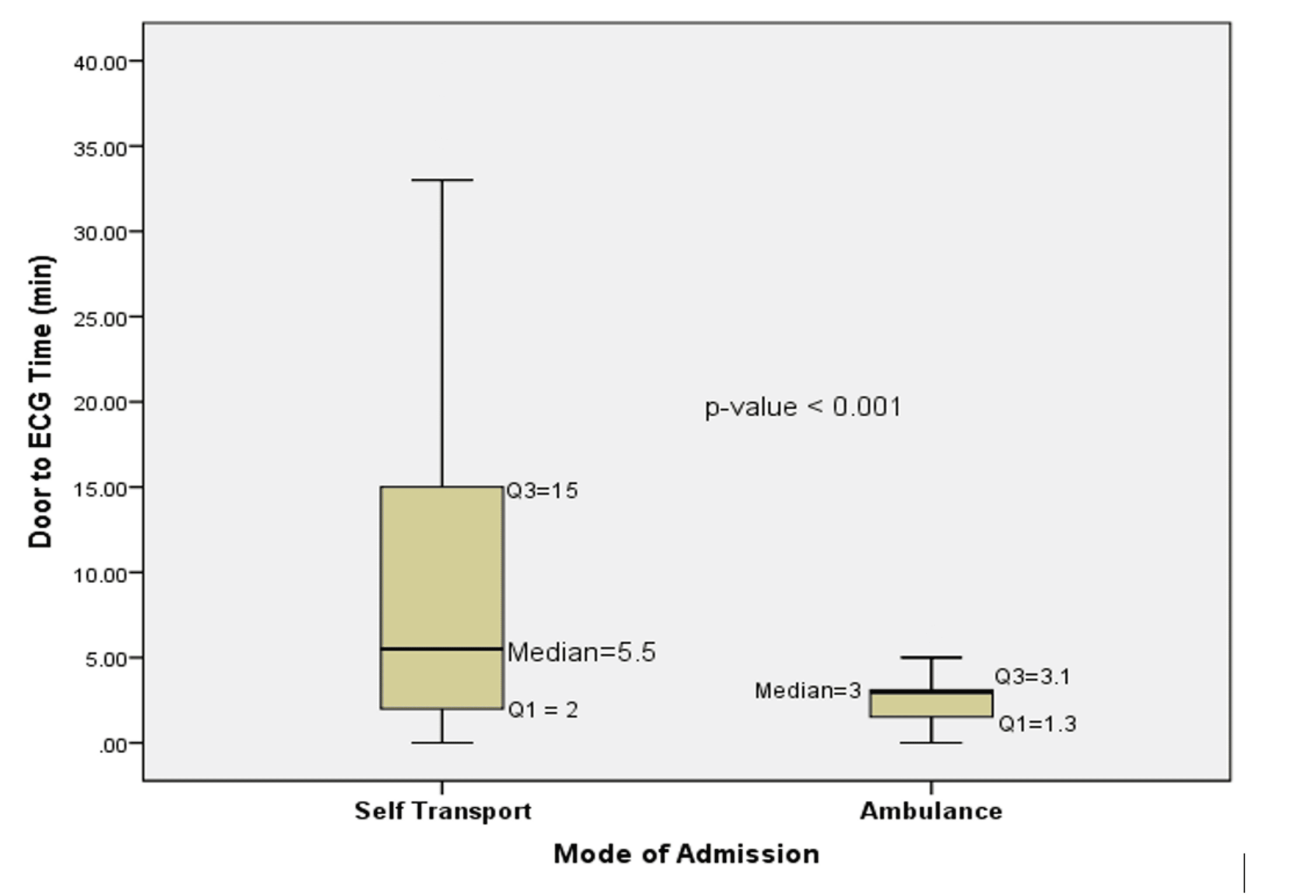
Door-to-ECG time in the EMS group compared to the self-transport group ECG: electrocardiogram; EMS: emergency medical services

Figure 2 compares the door-to-ECG time between self-transport and EMS patients. The median time for self-transport patients was significantly high at 5.5 minutes (IQR: 2-15), while for EMS patients, it was 3 minutes (IQR: 1.3-3.1). The difference was statistically significant (p<0.001).

**Figure 3:**
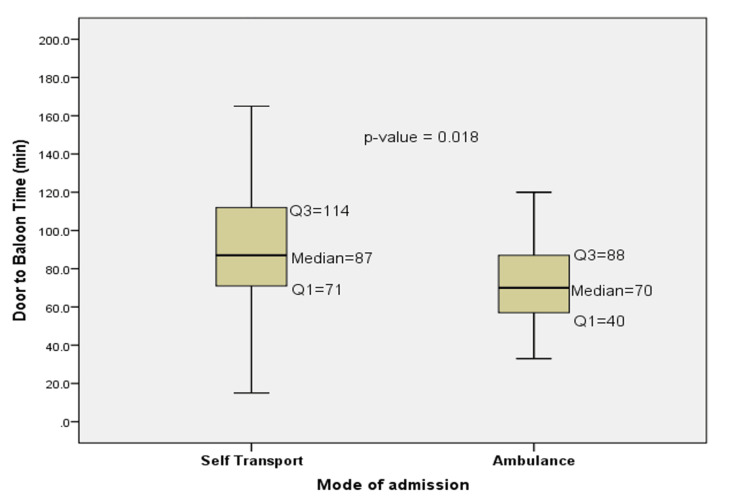
D2B time in the EMS group compared to the self-transport group D2B: door-to-balloon

Figure 3 compares the D2B time between self-transport and EMS patients. The median time for self-transport patients was significantly high at 87 minutes (IQR: 71-114), while for EMS patients, it was 70 minutes (IQR: 40-88). The difference was statistically significant (p=0.018).

Table 3 compares the clinical outcomes of patients based on their mode of transport. Ventricular tachycardia or ventricular fibrillation occurred in eight (4.7%) of self-transport patients and four (12.5%) of EMS patients. The difference was not statistically significant (p=0.08). A higher proportion of EMS patients (3, 9.4%) received interventions before catheterization compared to self-transport patients (7, 4.1%). The difference was not statistically significant (p=0.2). CPR was performed on nine (5.2%) of self-transport patients and six (18.8%) of EMS patients. The difference was statistically significant (p=0.007). The median peak troponin I levels were 46 (IQR: 18.4-116.6) in self-transport patients and 72 (IQR: 22.4-116.6) in EMS patients. The difference was not statistically significant (p=0.62). The median ejection fraction was 46% (IQR: 35-53%) in self-transport patients and 47% (IQR: 43-54%) in EMS patients. The difference was not statistically significant (p=0.41). The median hospital stay was two days (IQR: 1-3) for self-transport patients and 2.5 days (IQR: 1-3) for EMS patients. The difference was not statistically significant (p=0.55). The survival rate was high in EMS patients (31, 96.9%) compared to self-transport patients (163, 94.7%). The difference was not statistically significant (p=0.72).

**Table 3:** Comparison of in-hospital clinical outcomes between the EMS group and the self-transport group (n=204) EMS: emergency medical services; VT: ventricular tachycardia; VF: ventricular fibrillation; PCI: percutaneous coronary intervention; CPR: cardiopulmonary resuscitation

	Mode of transport		P-value
	Self-transport (n=172)	EMS (n=32)	
Episode of VT/VF	8 (4.7%)	4 (12.5%)	0.08
Any intervention done before PCI	7 (4.1%)	3 (9.4%)	0.2
CPR administered	9 (5.2%)	6 (18.8%)	0.007
Peak troponin I median (Q_1_-Q_3_)	46 (18.4-116.6)	72 (22.4-116.6)	0.62
Ejection fraction post-PCI median (Q_1_-Q_3_)	46% (35-53%)	47% (43-54%)	0.41
Length of hospital stay (in days) median (Q_1_-Q_3_)	2 (1-3)	2.5 (1-3)	0.55
Survived	163 (94.7%)	31 (96.9%)	0.72
Died	9 (5.3%)	1 (3.1%)	0.7

Table 4 presents the results of a multivariate logistic regression analysis to explore variables associated with the mode of transport (self-transport vs. EMS). The reference group for odds ratios is the EMS group. Length of hospital stay was not significantly associated with self-transport cases (OR=0.976; 95% CI: 0.89-1.07; p=0.614). For door-to-ECG time, the odds ratio of 1.212 suggests an association between longer door-to-ECG times and the use of self-transport; the result is statistically significant (p=0.003). In other words, for every additional minute in door-to-ECG time, the likelihood of a patient using self-transport increases by approximately 21.2% compared to EMS use. This result is statistically significant (p=0.003). For D2B time, the odds ratio of 1.011 suggests a weak association between longer D2B times and the use of self-transportation. In other words, for every additional minute in door-to-ECG time, the likelihood of a patient using self-transport increases by approximately 1.1% compared to EMS use. This result is not statistically significant (p=0.139). For deaths, the odds ratio of 4.01 suggests that patients who died were approximately four times more likely to have used self-transport compared to EMS use. However, the wide confidence interval (0.29-54.8) and the non-significant p-value (p=0.298) indicate that this result is not statistically reliable.

**Table 4:** Multivariate logistic regression analysis for variables associated with mode of transport D2B: door-to-balloon; ECG: electrocardiogram

	Odds ratio	95% CI	P-value
Length of hospital stay (in days)	0.976	0.89-1.07	0.614
D2B time	1.011	1-1.03	0.139
Door-to-ECG time	1.212	1.07-1.38	0.003
Died	4.01	0.29-54.8	0.298

## Discussion

The primary goal of the study was to explore the variations in D2B, door-to-ECG time, and clinical outcomes between patients with STEMI and NSTEMI who received primary PCI and arrived at the hospital through self-transportation compared to those who were transported by EMS. The demographic analysis showed no significant differences in gender, age, or nationality between self-transport and EMS groups. However, the clinical characteristics revealed that a higher proportion of EMS patients had comorbidities such as diabetes (84 (65.6%) vs. 21 (48.8%)) and hypertension (76 (56.3%) vs. 11 (44.2%)), although these differences were not statistically significant. This aligns with previous studies that have shown that patients with more severe or complex medical histories are more likely to utilize EMS [3].

The door-to-ECG time is a critical metric in the management of STEMI patients, as it represents the time from hospital arrival to the acquisition of an ECG, which is essential for diagnosing STEMI. In this study, the median door-to-ECG time was significantly shorter among EMS-transported patients (3 minutes) than among those who self-transported (5.5 minutes), likely due to expedited ECG acquisition facilitated by prehospital ECGs and early activation of the hospital STEMI team, which streamlines ED triage. Diercks et al. found that prehospital ECGs were associated with shorter door-to-ECG times and improved adherence to guideline-recommended treatment times [24]. This is particularly important because early ECG acquisition is a key step in the STEMI care pathway, as it triggers the activation of the PCI team. Patients who self-transport to the hospital often experience delays in receiving an ECG due to the need for triage and initial assessment in the ED. Ting et al. [16] found that self-transport patients faced delays in ECG acquisition compared to EMS patients, primarily due to the lack of prehospital ECG and the need for ED staff to prioritize patients based on initial presentation. Small delays in ECG acquisition can have a cumulative effect on overall treatment times, particularly when combined with other delays in the STEMI care pathway. Bradley et al. [25] found that shorter door-to-ECG times were associated with shorter D2B times, which are directly linked to improved patient outcomes.

The D2B time is one of the most critical metrics in STEMI care, representing the time from hospital arrival to the restoration of coronary blood flow via PCI. The American Heart Association and American College of Cardiology recommend a D2B time of less than 90 minutes, as shorter times are associated with reduced mortality and improved myocardial salvage [3]. In this study, the median D2B time was significantly longer for self-transport patients (87 minutes) compared to EMS patients (70 minutes). This finding is consistent with the previous research. Callachan et al. [26] found in their study that patients who self-transported experienced a longer D2B time, with a median of 81 minutes (IQR: 64-105 minutes). This was significantly longer compared to the median D2B time for patients arriving via EMS, which was 70 minutes (IQR: 48-89 minutes). The results of this study are in line with prior research indicating that EMS transport is associated with shorter symptom-onset-to-hospital arrival and D2B times [10,12]. Rathore et al. [13] found that every 15-minute reduction in D2B time was associated with a significant reduction in in-hospital mortality. This highlights the importance of minimizing D2B time to improve survival and reduce complications in STEMI patients. The shorter D2B time in EMS patients in this study likely contributed to the comparable survival rates between the two groups, despite the higher incidence of CPR in EMS patients. This suggests that timely reperfusion therapy can mitigate the risks associated with more severe initial presentations.

The study found that CPR was performed more frequently in EMS patients (6, 18.8%) compared to self-transport patients (9, 5.2%), which was statistically significant (p=0.007). This could indicate that EMS patients were more likely to experience severe complications such as cardiac arrest en route to the hospital, necessitating CPR. However, the survival rates were similar between the two groups (194 (94.7%) for self-transport vs. 31 (96.9%) for EMS), suggesting that despite the higher incidence of CPR in EMS patients, the overall outcomes were comparable. This finding is consistent with studies that have shown that while EMS transportation may be associated with more severe initial presentations, it does not necessarily translate into worse outcomes due to timely interventions [13]. A study from Miedema et al. [27] reported a similar death rate (6.2% for self-transport vs. 3.6% for EMS).

The multivariate analysis revealed that longer door-to-ECG times were associated with self-transport (OR=1.212; p=0.003). The odds ratio for D2B time (OR=1.011) was not statistically significant, suggesting that while EMS transportation may reduce D2B time, other factors such as hospital processes and patient characteristics may also play a role. Although the relation between D2B time and self-transportation was not statistically significant, the trend suggests that self-transporting patients may experience longer delays in receiving PCI. The lack of statistical significance in this study could be due to the relatively small sample size of EMS-transported patients (n=32), which limits the power to detect significant differences. The odds ratio for death (OR=4.01) was not statistically reliable due to the wide confidence interval, indicating that further research with a larger sample size is needed to explore this relationship.

Application

Our study highlights the continued underutilization of EMS, underscoring the need to investigate the underlying factors contributing to this gap, given that timely intervention is critical in MI and that EMS plays a pivotal role in minimizing D2B time and door-to-ECG times.

Limitations and recommendations

This study is exploratory in nature and has several limitations. First, its retrospective design and relatively small sample size, particularly in the EMS group (n=32), may limit statistical power and generalizability. Second, because our institution is one of several PCI-capable centers in the region and our hospital-based EMS does not provide direct community coverage, these factors likely influenced overall patient volume and the proportion of EMS-transported patients. Third, variables such as traffic conditions, distance from the hospital, and patient-level decision-making were not captured, all of which may affect the mode of transport and downstream clinical outcomes. Future studies should employ larger, prospective, multicenter designs to more robustly evaluate the association between mode of transport and clinical outcomes among patients undergoing primary PCI.

## Conclusions

This study demonstrates that EMS transportation is associated with shorter door-to-ECG and D2B times in patients. While the survival rates were similar between the two groups, the higher incidence of CPR in EMS patients suggests that these patients may have more severe initial presentations.
